# Study on Microstructure and Texture of Fe-3%Si Ultra-Thin Ribbons Prepared by Planar Flow Casting

**DOI:** 10.3390/ma17194893

**Published:** 2024-10-05

**Authors:** Jiangjie Xu, Ning Zhang, Yang Tu, Li Meng, Xiaozhou Zhou, Chengzhou Niu

**Affiliations:** Metallurgical Technology Institute, Central Iron and Steel Research Institute Co., Ltd., Beijing 100081, China; jiejie051618@163.com (J.X.); ty0171123688@163.com (Y.T.); zhouxiaozhou@cisri.com.cn (X.Z.); niuchengzhoucall@163.com (C.N.)

**Keywords:** planar flow casting (PFC), ultra-thin non-oriented silicon steel, temper rolling, deformation stored energy, crystal plasticity finite element (CPFE), {001} texture

## Abstract

In this paper, Fe-3%Si ultra-thin ribbons prepared by the planar flow casting (PFC) technique were subjected to temper rolling and annealing treatments. The microstructure and texture evolution during this process were examined through experimental measurements coupled with crystal plasticity finite element (CPFE) simulation to assess the feasibility of preparing ultra-thin non-oriented silicon steel using PFC ribbons. The results indicate that the PFC ribbons exhibit a significant columnar crystal structure, and {001}-oriented grains comprise over 30%. After being annealed, the grains with different orientations grew uniformly, the texture components were basically unchanged, and the {001} texture was well preserved. When annealing was carried out after temper rolling with a reduction rate of 7%, uneven grain growth was observed, and the growth tendency of the {001} grains, especially, surpassed that of the {111} grains, with an elevated temperature which peaked at 950 °C, where the proportion of {001} grains was maximal. When being annealed after temper rolling to 15%, grains of other orientations showed significant growth at each temperature, while the {001} grains did not show an obvious growth advantage. Utilizing the CPFE, the deformation-stored energy distribution of each characteristic-oriented grain was simulated, and it was shown that compared to the 15% rolling reduction rate, the deformation-stored energy accumulation of {001}-oriented grains after being rolled to 7% reduction was significantly lower than that of {111}-oriented grains. It suggests that the larger stored energy difference makes {001} grains show a stronger growth advantage based on the SIBM mechanism during annealing, after being rolled with a reduction rate of 7%. Overall, for the synergistic optimization of microstructure and texture, rolling with a 7% reduction rate followed by annealing at 950 °C in a hydrogen atmosphere is most advantageous.

## 1. Introduction

Non-oriented silicon steel is the core material for manufacturing motor cores, which is widely used in large, medium, and small generators, small motors, and other power electronic equipment. Its magnetic properties greatly affect the efficiency of power electronic equipment [1,2]. The decrease in silicon steel sheet thickness can significantly reduce the high-frequency iron loss, so as to meet the requirements of industrial development for high-frequency and low-loss core products. To produce ultrathin non-oriented silicon steel, the conventional rolling technique has the disadvantages of complexity, extended duration, and high energy consumption [3,4,5]. To achieve the carbon peak and carbon neutrality, the advancement of efficiency, energy-saving, and carbon-reducing production technologies will become the predominant trend in the future.

The planar flow casting (PFC) technique has inherent advantages in fabricating ultra-thin non-oriented silicon steel ribbons. Firstly, it is characterized by a short process flow and low energy consumption. The near-net-shape thin ribbons produced by the planar flow casting (PFC) technique require only temper rolling with a small reduction rate, then applying heat treatment to optimize microstructure and texture, thereby improving the magnetic properties. Meanwhile, the PFC ribbons have a certain ratio of columnar crystal structure by rapid solidification, so there are a large number of {001} (<001>//ND)-oriented grains. For Fe-3%Si steel with a body-centered cubic (BCC) structure, the easiest magnetization direction is the <001> direction, so a {001} texture can effectively improve the magnetic induction of the product [1]. Meanwhile, the PFC ribbons’ thickness advantage also directly reduces the energy consumption caused by the subsequent rolling [6,7].

It has been demonstrated that temper rolling and annealing treatment led to the transformation of microstructure and texture in silicon steel [8,9]. The magnetic properties of non-oriented silicon steel could be improved by enhancing grain size and uniformity, as well as strengthening the favorable texture. During temper rolling, it is suggested that the deformation-stored energy in different oriented grains is distinct, and the difference will affect the development of texture in the subsequent annealing process [10,11,12].

Since the initial grain size of non-oriented silicon steel is only a dozen micron, it is difficult for conventional experimental methods to accurately track the grain evolution during cold rolling. Therefore, this paper uses the crystal plasticity finite element simulation (CPFE) to study the temper rolling behavior of the PFC ribbons. Chen et al. [13,14,15,16,17] used CPFE simulation to study the microstructure changes during the rolling process of a Cu ultra-thin strip in detail, including the stress–strain distribution of grains, the activation of a slip system and the orientation transformation. Chu et al. [18] discussed the influence of the initial orientation structure on orientation change and deformation-stored energy during the cold rolling of non-oriented silicon steel by means of CPFE and an experiment. It is found that the energy storage accumulation rate of grains with different initial orientations during cold rolling deformation is different, and even if the different initial orientations are transferred to the same deformation orientation, the strain energy storage accumulation will also be different.

The annealing treatment also has a significant effect on the magnetic properties of the PFC ribbons. Liu et al. [19] studied the effect of annealing at different temperatures on the microstructure of PFC Fe-6.5%Si ribbons. They discovered that the optimal annealing temperature for magnetic properties was 1100 °C. Cheng et al. [20] also found that annealing treatment can effectively improve the magnetic properties of high silicon steel PFC ribbons. However, the related research on preparing Fe-3%Si ultra-thin non-oriented silicon steel ribbons using the PFC technique is still in the nascent stage.

Silicon can increase the electrical resistivity and reduce the iron loss [1]. For Fe-3%Si ultra-thin non-oriented silicon steel, it could be suitable for applications with higher magnetic induction requirements because of its higher saturation induction density (*B*s) compared with Fe-6.5%Si steel. Considering the easy <001> magnetization direction and the hard <111> magnetization direction, in the case of ultra-thin silicon steel production based on PFC, special attention needs to be paid to retaining/increasing the proportion of {001}-oriented grains and reducing the proportion of {111}-oriented grains during the temper rolling and following annealing, so as to improve the magnetic induction; meanwhile, a reasonable grain size increase is necessary for reducing iron loss [1,21]. This paper investigates the influence of temper rolling and annealing on the microstructure and texture of Fe-3%Si ultra-thin ribbons prepared via PFC technique, providing a foundation for determining optimal process parameters and magnetic performance enhancement strategies, thereby generating new insights for the preparation of ultra-thin non-oriented silicon steel ribbons utilizing PFC technique.

## 2. Materials and Methods

### 2.1. Experimental Materials and Characterization Methods

**Figure 1:**
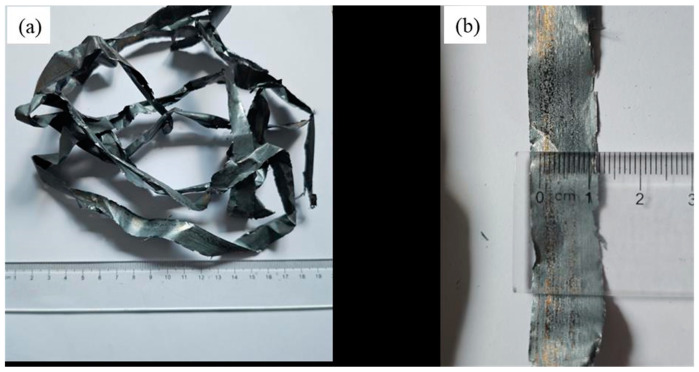
Different angle pictures of planar-flow-casted Fe-3%Si ribbons (**a**,**b**).

**Figure 2:**
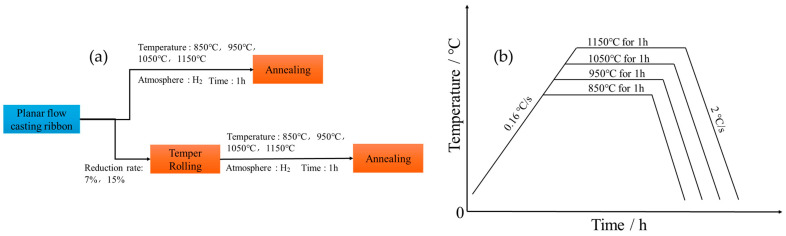
(**a**) Process flow diagram, and (**b**) heat treatment diagram.

A Fe-3.0%Si ingot with a weight of 25 kg was synthesized through induction melting using a mixture of pure Fe (99.9 wt%) and Si (99.99 wt%) in an argon atmosphere. The ingot was cut into small pieces of 1 kg and put into a quartz crucible with a diameter of 100 mm. It was then melted at 1650 °C using high-frequency induction heating. The molten alloy was then ejected onto a high-speed rotating water-cooled copper wheel through a boron nitride nozzle and rapidly solidified to form the PFC ribbons, as shown in Figure 1 Its size is approximately 10 mm wide and 0.065 mm in thickness. The PFC ribbons exhibit low thickness but poor flatness. Temper rolling with a 7% reduction rate was performed on the PFC ribbons, and temper rolling with a 15% reduction rate was conducted on a PFC ribbons with similar microstructural characteristics [21], followed by identical annealing treatment for both materials. Meanwhile, PFC ribbons were also annealed without being rolled to facilitate a comparison. The annealing temperatures employed were 850 °C, 950 °C, 1050 °C, and 1150 °C, respectively. The sample was placed into the furnace after the appropriate temperature was reached. The annealing time was set to 1 h, and the protective atmosphere utilized was dry, pure hydrogen (H_2_). The detailed process workflow is depicted in Figure 2.

The silicon steel ribbons at each processing stage were precisely cut into samples with dimensions of 10 mm (rolling direction, RD) × 5 mm (transverse direction, TD), and the samples were securely clamped using fixtures. Following grinding and mechanical polishing, the longitudinal sections were subjected to etching using a 4% nital solution. The rolling surface was initially polished using sandpaper and electropolished with 15% perchloric acid alcohol solution. The samples were collected and analyzed using an HKL Nordlys Max3 EBSD (UK High Wycombe Oxford Instrument Technology Co., Ltd., Oxford, UK) entsdetector installed on a Gemini SEM 500 (Carl Zeiss Management Co., Ltd., Oberkochen, Germany) field emission scanning electron microscope, with data processed through Oxford Instruments Channel 5 software (version 5.11.20405.0) to obtain orientation imaging, orientation distribution functions (ODF), and grain size measurements. To ensure the statistical robustness of the data, each sample was tested across 5–10 distinct regions, with the representative data presented in the text. Notably, the permissible deviation angle for each grain orientation was set to 20°.

### 2.2. Finite Element Simulation of Crystal Plasticity

**Figure 3:**
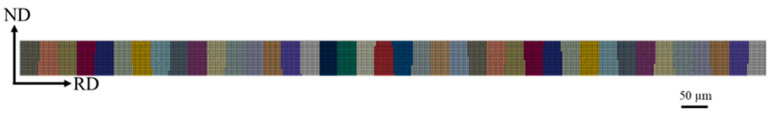
Reconstructed microstructure.

In this paper, the crystal plastic finite element method is used to simulate the deformation behavior of the PFC ribbon under different temper-rolling conditions. The constitutive model and simulation parameters the same as those detailed in the paper of Tu [22]. The simulation software is ABAQUS (version 2016). Because the PFC ribbon is basically unchanged along the transverse direction, thinned along the normal direction and elongated along the rolling direction during the temper-rolling leveling process, it is simplified as plane strain deformation in the simulation process. The grain morphology is simulated based on the Voronoi method, and the specific orientation information is given to each grain. The calculated model size is 0.065 mm × 3.5 mm, and the total number of grids is 21,220 units. The illustrational microstructure is shown in Figure 3. The different colors in the figure represent different orientations, and one grain is an aggregate of units with the same orientation.

## 3. Results

### 3.1. Microstructure and Texture of Planar-Flow-Casted Ribbons

**Figure 4:**
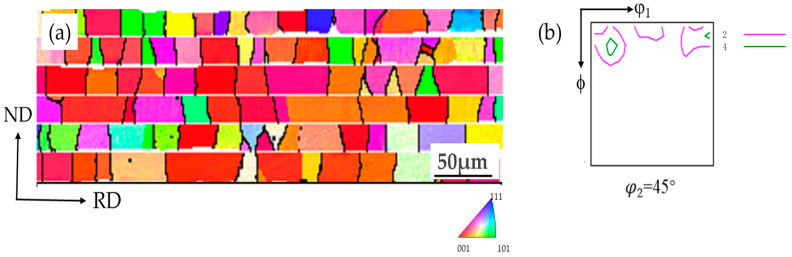
The microstructure and texture of planar-flow-casted ribbons. (**a**) EBSD inverse pole figure (IPF) map of microstructure, (**b**) φ_2_ = 45° section of ODF. (The different colors in the figure represent different orientations).

As depicted in Figure 4, this represents a typical lateral structural texture diagram of the PFC ribbons. The illustration shows that the initial microstructure of the ultra-thin non-oriented silicon steel ribbons fabricated via PFC exhibit a multitude of columnar grains spanning the entire thickness. The long axis of each grain predominantly aligns with the normal direction of the ribbons, with a deviation angle generally within 20°. The grains of each orientation are relatively uniform, and the average grain size is about 24.2 μm. The directionally solidified PFC ribbon exhibits a pronounced {001} (<001>//ND) texture, characterized by a significant proportion of {001}-oriented grains, constituting approximately 36.0%, whereas the {111}-oriented grains, which are detrimental to magnetic properties, constitute about 10.2%. This observation suggests that this study’s PFC ribbons possess a high initial magnetic induction.

### 3.2. Microstructure and Texture Changes during the Direct Annealing of Planar-Flow-Casted Ribbons

**Figure 5:**
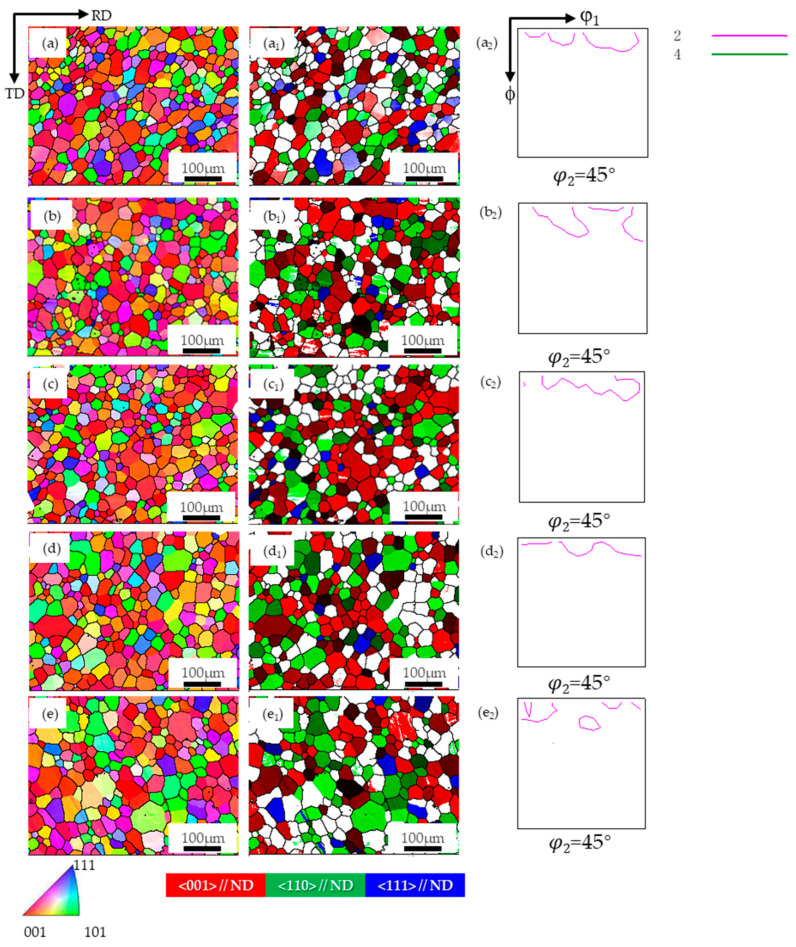
The microstructure and texture of planar-flow-casted ribbons after annealing at different temperatures. (**a**): initial state, (**b**) 850 °C, (**c**) 950 °C, (**d**) 1050 °C, (**e**) 1150 °C; in the map, (**a**–**e**): EBSD inverse pole figure (IPF) map of microstructure, (**a_1_**–**e_1_**): Grain distribution of the {001}, {110}, and {111} orientations (**a_2_**–**e_2_**): φ_2_ = 45° section of ODF.

**Figure 6:**
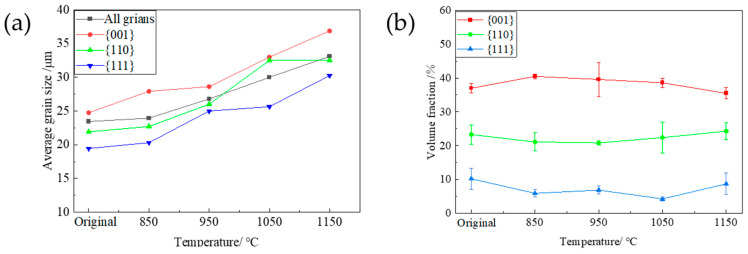
The average grain size distribution and the proportion of typical texture components after annealing at different temperatures. (**a**): Average grain size diagram of typical texture components; (**b**) The proportion of {001}-, {110}- and {111}-oriented grains.

Figure 5 shows the typical orientation imaging (IPF) and ODF images (φ_2_ = 45° section) of the PFC ribbons at different annealing temperatures (850 °C, 950 °C, 1050 °C, 1150 °C) during direct annealing. The average grain size and composition ratio of typical components were obtained by statistical analysis of multiple parallel data sets. The results are shown in Figure 6. It is observed that the average grain size increases from the initial 24 μm to 33.1 μm at 1150 °C, and the overall growth rate is less than 50%.

The average grain size oriented in the {001}, {110}, and {111} directions also increases uniformly, with a comparable degree of growth. It can be inferred that the surface energy does not considerably influence the grain growth kinetics of different orientations during the annealing process [19,21]. The average grain size and grain fraction at each temperature follow a descending order, namely {001}-oriented grains, {110}-oriented grains, and {111}-oriented grains. Moreover, the fraction of grains in each orientation remained relatively constant throughout the annealing process, implying that no significant texture transformation occurred, and a dominant {001} texture was consistently exhibited, suggesting that the favorable {001} texture was preserved during annealing.

### 3.3. Effect of Temper Rolling and Annealing on the Microstructure and Texture of Planar-Flow-Casted Ribbons

**Figure 7:**
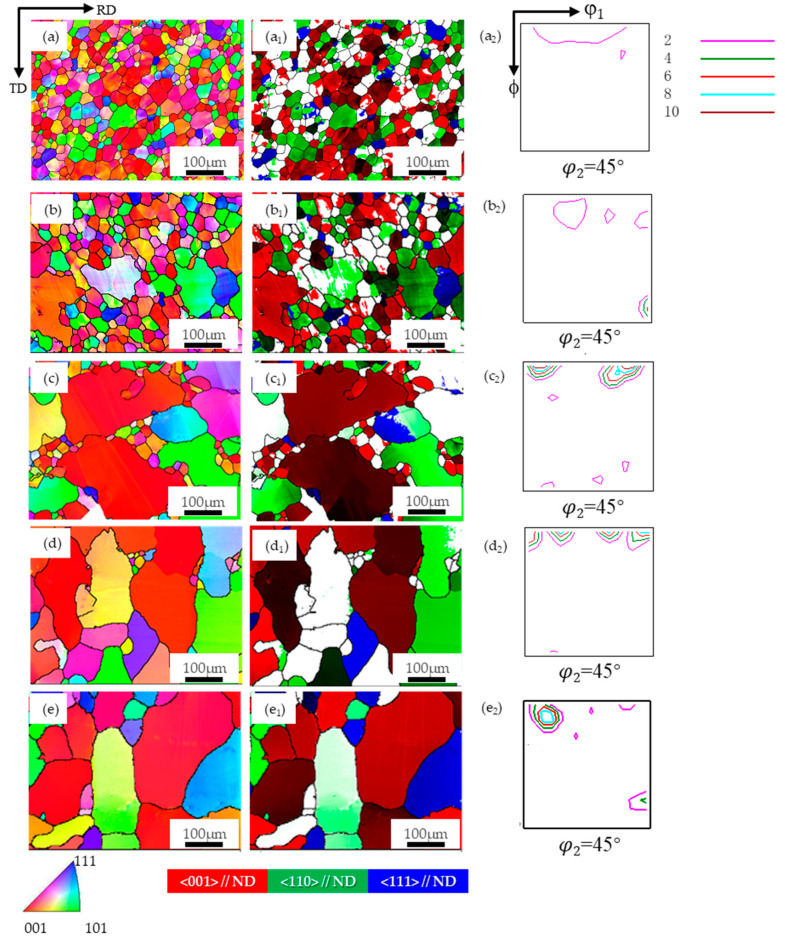
The microstructure and texture of planar-flow-casted ribbons at different annealing temperatures after temper rolling with a 7% reduction rate. (**a**): 7% rolled state, (**b**) 850 °C, (**c**) 950 °C, (**d**) 1050 °C, (**e**) 1150 °C; in the map, (**a**–**e**): EBSD inverse pole figure (IPF) map of microstructure, (**a_1_**–**e_1_**): Grain distribution of the {001}, {110}, and {111} orientations, (**a_2_**–**e_2_**): φ_2_ = 45° section of ODF.

**Figure 8:**
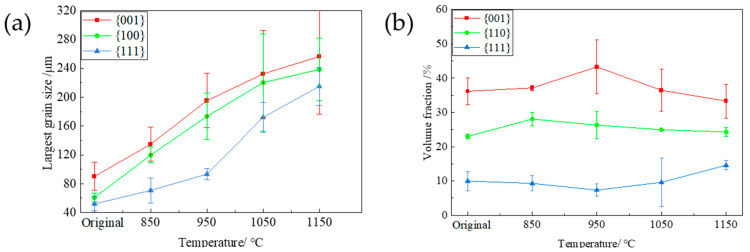
The grain size distribution and the proportion of typical texture components at different annealing temperatures after 7% reduction rolling of planar-flow-casted ribbons. (**a**) The largest grain size diagram of each typical texture component; (**b**) the proportion of {001}-, {110}-, and {111}-oriented grains.

Figure 7 depicts the the typical orientation imaging diagram (IPF), special orientation imaging diagram (<001>//ND, <110>//ND, and<111>//ND), and ODF (φ_2_ = 45° cross-section) of the PFC ribbons at different annealing temperatures (850 °C, 950 °C, 1050 °C, 1150 °C) after rolling at a 7% reduction rate. The largest grain size and composition ratio of different typical components are obtained by observing and counting multiple parallel data sets. The results are shown in Figure 8. As depicted in Figure 7, the texture alteration of the as-rolled structure is minimal owing to the slight deformation following the temper rolling of the PFC ribbons with a 7% reduction rate. After annealing, the grains grow unevenly, and there are still a certain number of fine grains at lower temperatures. The uniformity of the microstructure is gradually optimized by the gradual annexation of small-sized grains as the annealing temperature increases. At the same time, due to the size advantage and high proportion of large-size grains, the average grain size at each temperature is larger than that of the samples treated by direct annealing. Figure 8 illustrates that the {001}-, {110}-, and {111}-oriented grains can grow to larger sizes as the annealing temperature increases; however, the largest grain size disparity between the {001}- and {111}-oriented grains peaks at 950 °C and subsequently diminishes. The evolution of the largest grain size can characterize the grain growth rate during annealing. In this experiment, the grain growth rate exhibits a clear initial orientation dependence, indicating that the growth capability of {001} grains surpasses that of {111} grains at lower temperatures. The disparity in growth capability initially increases and then decreases with rising annealing temperature, peaking at 950 °C. Consequently, the proportion of {001} grains is highest at this temperature, while the proportion of {111} grains is lowest. Therefore, an annealing temperature of 950 °C under this system is optimal for the texture optimization of PFC ribbons, yielding the strongest {001} texture.

In Figure 7, it is evident that a pronounced {001} texture is consistently displayed across all annealing temperatures. Notably, a prominent Goss texture emerges at an annealing temperature of 950 °C. The preferential growth of the {001} texture and Goss texture is associated with their low Taylor factor value (~2.1), in contrast to the Taylor factor value of {111}-oriented grains, which ranges from 3.0 to 3.3 [6,23]. It is generally considered that the oriented grains with low Taylor factor accumulate less deformation-stored energy during the deformation process. The oriented grains with low deformation-stored energy grow before other grains after temper rolling and annealing, and the grain growth with high deformation-stored energy is swallowed by grain boundary migration; that is, the deformation-induced grain boundary migration mechanism (SIBM) [24,25,26], generally, in comparison to the initial state, the {001}-oriented grains and the {110} grain size, which include the Goss component, exhibit enhanced growth capabilities and increased proportions at annealing temperatures of 850 °C and 950 °C, which is consistent with the above analysis. However, it is important to note that the {111}-oriented grains and other atypical oriented grains also grow significantly when annealed at 1050 °C and 1150 °C; this phenomenon also results in the weakening of the {001} texture at elevated temperatures, which may due to the general enhancement of dislocation movement and grain boundary migration ability at high temperatures, so that the growth advantage of grains grown with a low energy storage advantage is no longer significant.

**Figure 9:**
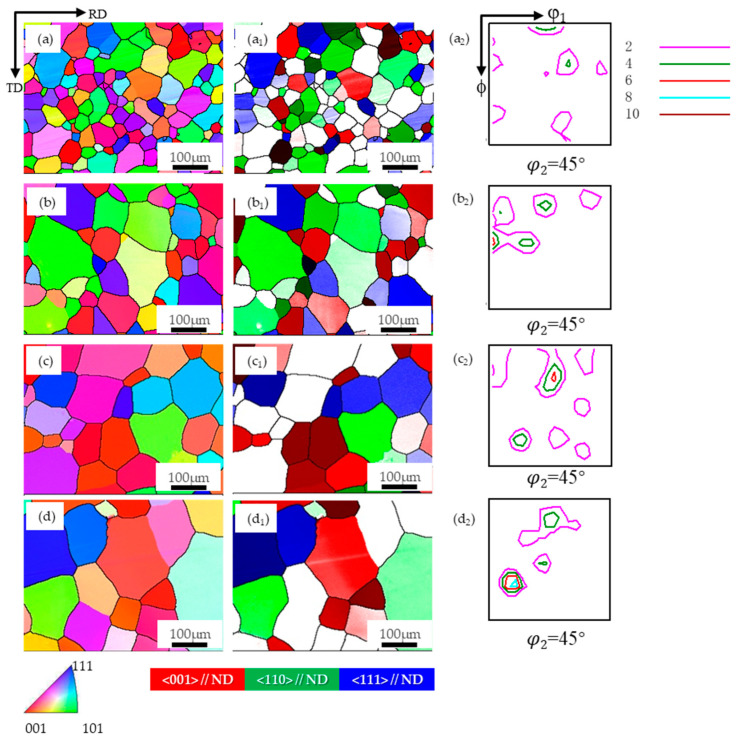
The microstructure and texture of planar-flow-casted ribbons at different annealing temperatures after temper rolling with 15% reduction rate. (**a**) 850 °C, (**b**) 950 °C, (**c**) 1050 °C, (**d**) 1150 °C; in the map, (**a**–**d**): EBSD inverse pole figure (IPF) map of microstructure, (**a_1_**–**d_1_**): Grain distribution of the {001}, {110} and {111} orientations, (**a_2_**–**d_2_**): φ_2_ = 45° section of ODF.

**Figure 10:**
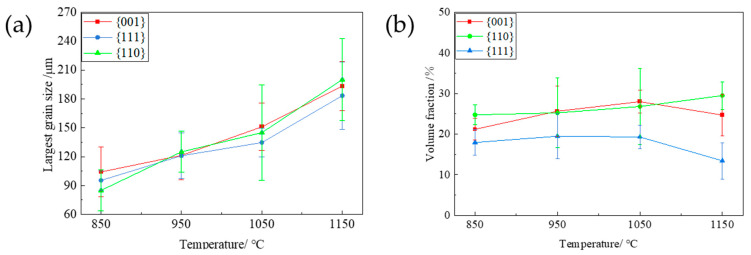
The grain size distribution and the proportion of typical texture components at different annealing temperatures after a 15% reduction rolling of the planar-flow-casted ribbon. (**a**) The largest grain size diagram of each typical texture component; (**b**) the proportion of {001}-, {110}-, and {111}-oriented grains.

In Zhang et al.’s [21] study, it was discovered that when a PFC Fe-3%Si ribbon, exhibiting comparable texture characteristics, was subjected to rolling at 15% reduction rate and annealing, the growth advantages of the {001} and Goss components with low Taylor factor values depicted in Figure 7 were not observed. To further explore the range of rolling reduction rates that can achieve texture optimization, this paper also performed annealing treatment at 850 °C, 950 °C, 1050 °C, and 1150 °C for 1h on the PFC ribbons after rolling at a 15% reduction rate. The corresponding texture evolution is shown in Figure 9 and Figure 10. It is evident that the grains exhibit more uniform and pronounced growth post-annealing, with the average grain size being markedly larger compared to direct annealing.

The largest grain sizes of {001}-, {110}- and {111}-oriented grains show a significant equivalent increase with the increase in temperature; that is, the {001} grains at each temperature do not show a significant growth advantage similar to the annealing treatment after 7% reduction rate rolling, which is different from other oriented grains. Additionally, the proportion of {111} grains at each temperature increases markedly, and the {001} texture is evidently weakened due to the growth of other atypical texture components. Through comprehensive analysis, although the grain size growth is better than that of the direct annealing sample, the average grain size at higher temperatures is equivalent to that at the 7% reduction rate, and it is not conducive to the retention and optimization of the {001} texture currently. Overall, for the synergistic optimization of microstructure and texture, it should be optimal for the PFC samples to be annealed after rolling at a 7% reduction rate. It can be speculated that for this material system, achieving {001} texture optimization via the SIBM mechanism requires controlling the rolling deformation within a lower range, and a more precise critical value necessitates further investigation in the future.

### 3.4. Differential Analysis of Grain Deformation Stored Energy with Different Characteristic Orientations

**Figure 11:**
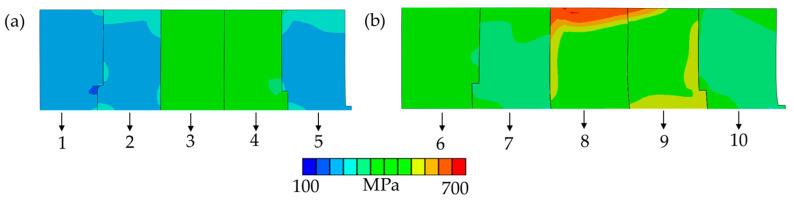
Mises stress of microstructure reconstructed by crystal plasticity finite element model (**a**): 7% reduction rate, the Mises stress distribution of grains 1–5 ({001}<100>, {001}<110>, {111}<110>,{111}<112>, {011}<100>) with different orientations in the reconstructed microstructure; (**b**): 15% reduction rate, the Mises stress distribution of grains 6–10 ({001}<100>, {001}<110>, {111}<110>,{111}<112>, {011}<100>) with different orientations in the reconstructed microstructure.

**Figure 12:**
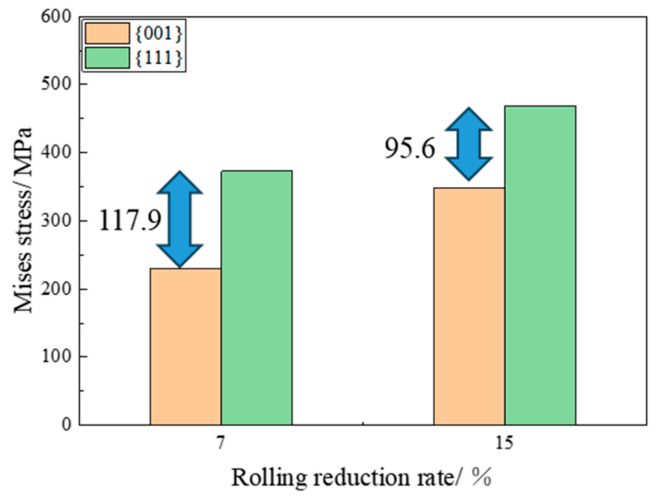
Average Mises stress of grains at different rolling reduction rates. (The arrows in the figure represent the difference value between {001} and {111} grans).

To elucidate the underlying mechanisms responsible for the disparity in grain growth capabilities among different orientations post rolling at varying rolling reduction rates, as illustrated in Figure 8 and Figure 10, this study employs a CPFE model grounded in phenomenological theory to simulate the distribution of Mises stress in grains with distinct orientations under different rolling reduction rates. The magnitude of the Mises stress indicates the level of deformation-stored energy [18,27,28]. In this study, we selected several typical texture components specifically, grains oriented to ({001} <100>, {001} <110>, {111} <110>, {111} <112>, {011} <100>) as the research subjects, and calculated the Mises stress distribution for each oriented grain. The representative data are presented in Figure 11. It is evident that the accumulation of deformation-stored energy during the rolling process significantly depends on the initial orientation. Under varying reduction rates, the deformation stored energy in {001} and Goss-oriented grains is found to be lower than that in {111}-oriented grains. The Mises stress value in Goss-oriented grains is basically the same as that in {001}-oriented grains, which aligns with the Taylor factor calculation results reported in the literature [6,23]. To enhance the statistics, the grain shape and adjacent relationship are randomly set for simulation calculation, and the average Mises stress of {001}- and {111}-oriented grains under different reduction rates is derived from the statistical analysis of multiple data sets, as shown in Figure 12. It is observed that as the reduction rate increases from 7% to 15%, the Mises stress values in each grain increase, the average Mises stress difference between {001} grains and {111} grains decrease, implying a decrease in the deformation-stored energy difference. It can be speculated that this diminished deformation-stored energy difference attenuates the driving force for the growth of {001}-oriented grains, subsequently leading to a reduction in their preferential growth capability.

## 4. Conclusions

(1) The Fe-3% Si ultra-thin non-oriented silicon steel ribbons prepared by planar flow casting (PFC) exhibit a significant columnar crystal structure, with {001}-oriented grains constituting over 30%. After annealing, the average grain size exhibits a slight increment with the rising annealing temperature. The grains of different orientations grow evenly, the proportion of grains of each orientation is basically unchanged, and the {001} favorable texture is well preserved.
(2) Uneven grain growth was observed when the planar-flow-casted (PFC) ribbons underwent annealing following temper rolling with 7% reduction. The growth capability of {001}-oriented grains surpassed that of {111}-oriented grains. The disparity in this growth capability initially increased and then decreased with rising annealing temperatures, and this difference peaked at 950 °C, leading to the highest proportion of {001} grains and the lowest proportion of {111}-oriented grains at this temperature. Following temper rolling with a 15% reduction rate and subsequent annealing, grains of each orientation exhibited significant growth at all temperatures. That is, the {001}-oriented grains did not have a pronounced growth advantage better than others, and the {001} texture is weakened while the {111} texture is strengthened. Overall, for the synergistic optimization of microstructure and texture, rolling at a 7% reduction rate followed by annealing at 950 °C in a hydrogen atmosphere is most advantageous.
(3) Based on crystal plasticity finite element (CPFE) simulations, it was found that the deformation stored energy of {001}- and Goss-oriented grains is lower than that of {111}-oriented grains at various reduction rates. At 7% reduction, the deformation stored energy accumulation of {001} grains is much less than that of {111} grains after rolling, and this marked stored energy difference contributes to the strong growth advantage of {001} grains during annealing based on SIBM mechanism.

## Data Availability

The data presented in this study are available on request from the corresponding author due to ongoing research and further analysis.
